# Return of the Biennial Circulation of Enterovirus D68 in Colorado Children in 2024 Following the Large 2022 Outbreak

**DOI:** 10.3390/v17050673

**Published:** 2025-05-05

**Authors:** Hai Nguyen-Tran, Molly Butler, Dennis Simmons, Samuel R. Dominguez, Kevin Messacar

**Affiliations:** 1 Department of Pediatrics, University of Colorado School of Medicine, 13001 E 17th Place, Aurora, CO 80045, USA; samuel.dominguez@childrenscolorado.org (S.R.D.); kevin.messacar@childrenscolorado.org (K.M.); 2 Children’s Hospital Colorado, 13123 E 16th Ave, Aurora, CO 80045, USA; molly.butler@childrenscolorado.org (M.B.); dennis.simmons@childrenscolorado.org (D.S.)

**Keywords:** enterovirus D68, surveillance, respiratory disease

## Abstract

Enterovirus D68 (EV-D68) caused large biennial cyclical outbreaks of respiratory disease and cases of acute flaccid myelitis from 2014 to 2018 in the USA. An anticipated outbreak did not occur in 2020, likely due to non-pharmaceutical interventions targeting the COVID-19 pandemic. A large respiratory disease outbreak occurred again in 2022, but uncertainty remained regarding if circulation of EV-D68 would return to the pre-pandemic patterns. We conducted prospective active surveillance of clinical respiratory specimens from Colorado children for EV-D68 in 2023 and 2024. A subset of residual specimens positive for rhinovirus/enterovirus (RV/EV) were tested for EV-D68 via a validated in-house EV-D68 reverse transcription–PCR assay. During epi weeks 18–44 in 2023, 525 residual specimens positive for RV/EV all tested negative for EV-D68. In 2024, during epi weeks 18–44, 10 (1.8%) of the 546 RV/EV-positive specimens were EV-D68-positive. The EV-D68-positive cases were predominantly young children (median age 4.8 years) receiving treatment with asthma medications. Following the 2022 EV-D68 outbreak, an anticipated outbreak did not occur in 2023. While EV-D68 was detected in 2024, the number of cases was not as significant as in prior outbreak years. Continued surveillance for EV-D68 will be important to understand the future dynamics of EV-D68 circulation and prepare for future outbreaks.

## 1. Introduction

Biennial cyclical outbreaks of enterovirus D68 (EV-D68) respiratory disease and acute flaccid myelitis (AFM) cases occurred in the USA and around the world from 2014 to 2018 [1,2,3,4,5,6,7,8,9,10,11,12,13]. An outbreak of EV-D68 did not occur in 2020, likely due to non-pharmaceutical interventions (NPIs) targeting the COVID-19 pandemic, which similarly impeded the circulation of other endemic respiratory viruses [14]. However, following the lifting of the COVID-19 pandemic restrictions, EV-D68 re-emerged once again, with increased circulation seen in Europe in 2021 and in the USA in 2022 [15,16,17]. Despite an increase in EV-D68 respiratory disease cases in 2022 in the USA, no increase in AFM cases was seen during this timeframe [17,18]. Following the large EV-D68 respiratory disease outbreak in the USA in 2022, uncertainty remained regarding the re-emergence of EV-D68 and if its circulation would return to its prior biennial cyclical outbreak patterns or levels. The objective of this study was to understand the epidemiology and circulation of EV-D68 in children from Colorado following the 2022 outbreak.

## 2. Materials and Methods

This study entailed prospective active surveillance of clinical respiratory specimens from children presenting for care at the Children’s Hospital Colorado (CHCO). The CHCO is a large academic, quaternary care hospital with a seven-state catchment area that primarily serves children in the Denver metropolitan area. In Colorado, we established a multimodal surveillance system for EV-D68 consisting of syndromic, clinical laboratory, and wastewater surveillance [17]. As part of this multimodal surveillance system, during typical EV-D68 circulation months (late summer–early fall), a subset of residual clinical respiratory specimens from primarily inpatient children at the CHCO that tested positive for rhinovirus (RV)/enterovirus (EV) on the BioFire^®^ Respiratory 2.1 (RP2.1) panel (bioMerieux, Salt Lake City, UT, USA) were subsequently tested for EV-D68 [17]. The CHCO uses a validated in-house EV-D68-specific reverse transcription–polymerase chain reaction (RT-PCR) assay that uses a primer–probe set designed to detect subclade B3 and previously circulating strains [17,19]. Given the uncertainty concerning when EV-D68 would circulate, the EV-D68 clinical laboratory surveillance began earlier in the year than the typical circulation months, beginning in May and extending through October in 2023 and 2024.

## 3. Results

**Figure 1:**
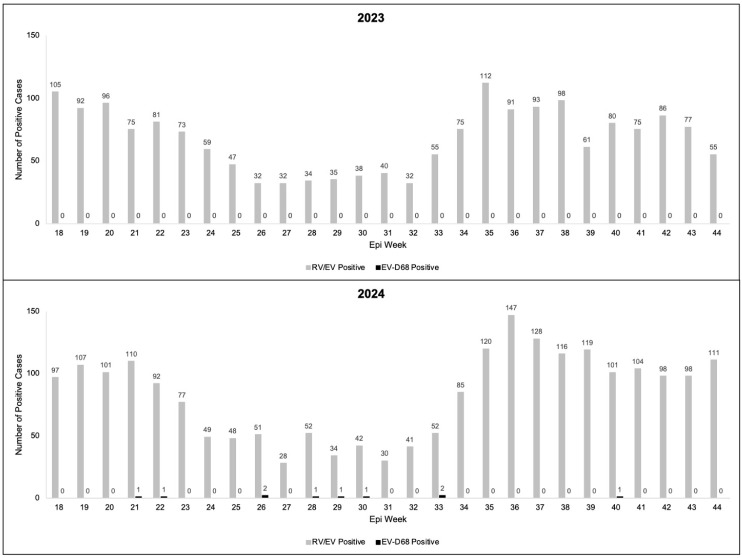
Enterovirus D68 (EV-D68)- and rhinovirus/enterovirus (RV/EV)-positive cases in children in Colorado by epi week in 2023 and 2024.

In 2023 and 2024, there were 5634 and 7248 clinical respiratory specimens sent for RP2.1 testing during epi weeks 18–44 (May–October), respectively. Of these specimens, 1829 (32.5%) and 2238 (30.9%) were positive for RV/EV in 2023 and 2024, respectively (Figure 1). In 2023, all of the 525 residual clinical respiratory specimens testing positive for RV/EV tested negative for EV-D68 (Figure 1).

During 2024, 546 residual clinical respiratory specimens positive for RV/EV were tested for EV-D68. Of the 546 tested, 10 (1.8%) were positive for EV-D68 (Figure 1). The median age of the children was 4.8 (range: 1.3, 10.7) years and 50% were males (Table 1). All of the EV-D68-positive children were admitted to the hospital and eight required intensive care unit (ICU) admission. All the EV-D68-positive children required supplemental oxygen support, with two requiring intubation. Though only four of the EV-D68-positive children had a history of asthma, eight were treated with albuterol, a medication typically reserved for asthma. Only one of the EV-D68-positive patients had a concurrent pathogen, *Mycoplasma pneumoniae*, detected on the RP2.1, and seven were treated with antibiotics due to concern about concurrent bacterial pneumonia. No confirmed AFM cases were seen in 2023 or 2024 in Colorado.

## 4. Discussion

Outbreaks of EV-D68 respiratory disease and cases of AFM result in significant resource strains for healthcare systems and associated morbidity for patients and families [20,21,22]. Following a large EV-D68 respiratory disease outbreak in 2022, with a lack of increase in AFM cases seen in the USA, uncertainty remained regarding if the EV-D68 circulation would return to its prior biennial cyclical pattern and if the decoupling seen with outbreaks of EV-D68 respiratory disease and AFM would persist. In Colorado, we did not see EV-D68 circulate in 2023, and while there were EV-D68 detections seen in our clinical laboratory surveillance in 2024, the number of cases was significantly lower compared to prior outbreak years and without associated AFM cases. Similar to previous outbreaks, the EV-D68-positive respiratory cases in 2024 tended to occur in young children with a median age of 4.8 years and a majority of cases had asthma-like respiratory disease presentations requiring medications typically used in the treatment of asthma.

The prior outbreak patterns of EV-D68 can be explained by serotype-specific immunity, with large outbreaks leading to a decrease in the susceptible population limiting the size of another outbreak until subsequent birth cohorts replenished the pool of susceptible individuals [23,24]. As seen during the COVID-19 pandemic, the typical circulation dynamics of many endemic respiratory viruses were disrupted and an expected outbreak of EV-D68 in 2020 did not occur [25]. In Colorado, our EV-D68 multimodal surveillance system provided timely and actionable data to allow us to recognize and prepare for the 2022 outbreak [17]. Following the 2022 outbreak, the patterns we saw in 2023 and 2024 in our EV-D68 clinical laboratory surveillance were consistent with what we saw in the other layers of our multimodal surveillance system in Colorado and what was reported across the USA [26]. Given the large outbreak in 2022 and the subsequent increase in population immunity, we suspect that there has not yet been sufficient time for the growth of a susceptible pool to lead to another large outbreak, but similar to other endemic respiratory viruses, we hypothesize the return to pre-pandemic levels and circulation dynamics in the coming years. However, changes in the virulence of circulating strains of EV-D68 could also affect the recent patterns seen and the future circulation dynamics [27].

Limitations of this study exist. As a single-center study, the results may not be fully generalizable; however, similar patterns of EV-D68 circulation in 2023 and 2024 have been reported across the USA [26]. Our EV-D68 RT-PCR assay is designed to detect subclade B3 and prior circulating strains. While subclade B3 has been the predominant subclade circulating in 2016, 2018, and 2022, if new strains have emerged, they may have been missed by our assay [17,27]. As a majority of the EV-D68-positive patients in 2024 also received antibiotics for potential bacterial pneumonia, the severity of the few EV-D68 infections we saw in 2024 may be confounded by the concurrent pneumonias in patients. Furthermore, our clinical laboratory surveillance only tests a subset of RV/EV-positive samples and utilizes testing of RP2.1 obtained from children presenting for care and ordered at the clinical discretion of the provider taking care of the patient. Therefore, our clinical laboratory surveillance likely underestimates the true burden of EV-D68 disease, particularly in patients with more mild presentations or asymptomatic cases that do not present for care.

Following the prior biennial cyclical outbreak patterns, no EV-D68 circulation was detected in Colorado in 2023. While circulation was detected in 2024, the number of detections for EV-D68 was not as significant as in prior outbreak years and was not associated with AFM cases. Continued surveillance of EV-D68 will be important to monitor for potential future outbreaks and determine if the circulation dynamics will return to pre-pandemic levels and patterns.

## Figures and Tables

**Table 1:** Demographics and clinical admission characteristics of the enterovirus D68 (EV-D68)-positive cases in Colorado in 2024.

	**EV-D68-Positive Cases** **(*N* = 10)**
Age (years)	
Median (IQR)	4.8 (3.4, 6.0)
Range	1.3, 10.7
Sex, n (%)	
Male	5 (50)
Female	5 (50)
Admission, n (%)	
Yes	10 (100)
No	0 (0)
Admission Length of Stay (days) *	
Median (IQR)	4 (2, 5)
Range	1, 22
Required Intensive Care Unit (ICU) Admission, n (%)	
Yes	8 (80)
No	2 (20)
ICU Length of Stay (days) *	
Median (IQR)	1 (1, 2.5)
Range	0, 7
Supplemental Oxygen Required, n (%)	
Yes	10 (100)
No	0 (0)
Maximum Oxygen Support Required, n (%) *	
Low-flow nasal cannula	1 (10)
Heated high-flow nasal cannula	1 (10)
Non-invasive positive pressure ventilation	5 (50)
Intubation	2 (20)
Unknown	1 (10)
History of Asthma, n (%)	
Yes	4 (40)
No	6 (60)
Medications Received, n (%)	
Albuterol	8 (80)
Steroids	7 (70)
Antibiotics	7 (70)

Abbreviation: IQR, interquartile range. * One patient was transferred to an outside hospital. The length of stay and maximum oxygen support were unknown for this patient.

## Data Availability

Data may be made available upon reasonable request to the corresponding author for investigators whose proposed use of the data has been approved by the COMIRB.
